# Neuroleptic Malignant Syndrome in Children with Autism Spectrum Disorder (ASD): A Case Report and Brief Review of Recent Literature

**DOI:** 10.3390/children8121201

**Published:** 2021-12-18

**Authors:** Stefano Berloffa, Claudia Dosi, Benedetta Tascini, Beatrice Fossati, Ilaria Lupetti, Gabriele Masi

**Affiliations:** 1 IRCCS Stella Maris, Scientific Institute of Child Neurology and Psychiatry, 56128 Pisa, Italy; stefano.berloffa@fsm.unipi.it (S.B.); dosiclaudia@gmail.com (C.D.); beatrice.fossati@fsm.unipi.it (B.F.); ilarial.89@otmail.it (I.L.); 2 Department of Woman’s and Children’s Health, Padua University Hospital, 35128 Padua, Italy; benedetta.tascini@aopd.veneto.it

**Keywords:** neuroleptic malignant syndrome (NMS), autism spectrum disorder (ASD), catatonia, pediatric

## Abstract

Neuroleptic malignant syndrome (NMS) is a rare, life-threatening, idiosyncratic adverse reaction to antipsychotic drugs. Despite the increasing rates in the prescription of antipsychotics in pediatric patients with autism spectrum disorder (ASD), little is known about the occurrence and hallmarks of NMS in this specific population. NMS appears to be part of the larger catatonia domain, based on the frequent relationship between ASD and catatonia, on the shared, when not overlapping, clinical features with malignant catatonia, and on the effectiveness of catatonia treatments on the NMS/MC symptoms. The intrinsic difficulties of exploring NMS in ASD in controlled studies accounts for the subsequent lack of available information. Based on recent reports and on our case report, clinical features of NMS in the pediatric ASD population appear to be the same as the non-ASD population. Further studies are needed to confirm these results.

## 1. Introduction

Neuroleptic malignant syndrome (NMS) is a rare, life-threatening, iatrogenic condition mainly associated with antipsychotic drug use, and is characterized by fever, altered mental status, muscle rigidity, and autonomic dysfunction. Despite an increased medical awareness, many aspects regarding its epidemiology, physiopathology, and nosology remain controversial, and the syndrome continues to be a clinical challenge.

The NMS was first described in 1960 by Delay and colleagues [1], after the introduction of haloperidol to the market in the late 1950s. When second-generation antipsychotics became available, hopes were high that they would be free from the risk of inducing NMS; unfortunately, most atypical antipsychotics (i.e., aripiprazole, risperidone, olanzapine, clozapine, and paliperidone), have been reported over the years to be associated with NMS, as well as typical AP (i.e., haloperidol, flupentixol, chlorpromazine, and periciazine) [2,3,4]. Moreover, other classes of drugs have been associated with NMS-like symptoms, such as lithium, carbamazepine, paroxetine, and metoclopramide [2,3,5], following unclear pathways. 

Traditionally, NMS is considered to be the result of dopaminergic D2 receptor antagonism or dopamine agonist withdrawal in the central nervous system and spinal cord [6,7], which leads to a cascade of reactions, primarily involving the thermoregulatory center of the hypothalamus, with subsequent hyperthermia, and the basal ganglia, which could explain the motor signs characteristic of this condition, such as rigidity, hypertonia, and tremor. A non-alternative model proposes the concomitant role of toxicity of the musculoskeletal fibers, supported by the response to dantrolene in NMS and typical antipsychotic drug effects on calcium regulation in skeletal muscular fibers [2]. 

Over the years, many attempts to standardize diagnostic criteria have been made [8,9], the most recent one by the DSM-5 [10]. NMS is heterogeneous in onset, presentation, course, and outcome. Clinical risk factors for NMS include psychomotor agitation, confusion, disorganized behavior, and catatonia, extrapyramidal signs such as akathisia, while possible pharmacological risk factors are higher dosages, fast titration, and parenteral injections [8,9]. No pathognomonic sign has ever been found. DSM-5 describes the most common clinical features, which include a positive history for dopamine antagonist use (usually within 72 h prior to symptom development), as hyperthermia, generalized rigidity, described as “lead pipe” in its most severe form, and other neurological and medical symptoms (e.g., tremor, sialorrhea, akinesia, dystonia, trismus, myoclonus, dysarthria, dysphagia, and rhabdomyolysis). They also include, in most cases, changes in mental status, characterized by delirium or altered consciousness (often an early sign), and autonomic instability (tachycardia, diaphoresis, blood pressure elevation or fluctuation, urinary incontinence, and tachypnea). The onset of symptoms varies from hours to days after drug initiation. Underlying pathophysiological mechanisms are still unclear. 

The first step in the diagnostic process is to rule out other etiologies including infections, toxin exposure, or metabolic or neurologic causes. Laboratory findings usually include leukocytosis, creatine kinase (CK) elevation of at least four times the upper limit of normal (up to 100,000 IU/L), and decreased serum iron concentrations. CK monitoring not only serves diagnostic purposes, but also for monitoring the condition, since levels of CK must decrease over time as the condition improves [2]. However, misleading high levels of CK may occur after intramuscular injections, restraints, intense physical activity, or dystonic reactions [11]. Furthermore, a massive asymptomatic CK elevation has been described during antipsychotic exposure in nonpsychotic, drug-naïve youth during treatment with second-generation antipsychotics. In these cases, a drug discontinuation should be considered only when possible signs of NMS or rhabdomyolysis are suspected (i.e., flu-like syndrome, fever, weakness, alteration of consciousness, muscle rigidity, tachycardia, hyper-/hypotension, and dark urine) [11].

Autism spectrum disorder (ASD) is a complex neurodevelopmental disorder characterized by persistent deficits in social communication and social interaction across multiple contexts, and restricted, repetitive patterns of behavior, interests, or activities [10]. Pharmacological treatments are commonly used in individuals with ASD to improve associated ASD symptoms such as irritability and agitation, and to treat co-occurring psychiatric conditions such as ADHD, anxiety, depression, bipolar disorder, psychotic symptoms, and other disorders. Although there are currently no rigorous evidence-based guidelines regarding psychotropic medications for children with ASD [12], several randomized controlled trials (RCTs) with antipsychotics in children with ASD have been conducted over the years, mostly risperidone and aripiprazole. Large RCT trials have shown results supporting that both medications are superior to the placebo in alleviating irritability (agitation, anger outbursts, and self-injurious behavior), stereotypy, and hyperactivity [13,14]. Few studies are also available concerning other second-generation antipsychotics, [13,15]. Among the first-generation antipsychotics, haloperidol is the most studied and the only one proven to be effective on behavioral symptoms in ASD, although associated with severe side effects [13]. These data have been confirmed by successive RCTs [14,16]. These studies have led to the approval by the U.S. Food and Drug Administration (FDA) and other European medical agencies of two antipsychotic medications, risperidone and aripiprazole, for the treatment of irritability in children with ASD [17].

Currently, there are no known contraindications to using common antipsychotic medications for children with ASD, although some experts believe that atypical responses (e.g., idiosyncratic, disinhibition, or paradoxical reactions) may be more common [12]. Inquiring about previous reactions to medications is often helpful, as may be beginning with lower dosages to observe and determine the child’s response to the medication [12]. But what do we know about NMS in autistic children?

Recent literature regarding autism and NMS mostly focuses on the association with catatonia and on the treatment with ECT [18,19], given the higher risk for developing catatonia in this population [18]. Clinical features and laboratory findings are comparable to those described in the non-ASD population, as summarized in Table 1, Table 2 and Table 3**.**

Data on other treatments for catatonia in children with ASD are still lacking, except for a recent case report including two patients successfully treated with clozapine [20].

## 2. Case Presentation

We describe the case of an 18-year-old boy presented with ASD associated with a mild intellectual disability (patient 5 in the tables). Informed consent was obtained from all subjects involved in the study. Regarding the familial load, the paternal uncle presents an anxiety disorder treated with a selective serotonin reuptake inhibitor.

The proband is the first child of unrelated and healthy parents. He attended school with support, had good global functioning and social relationships with classmates, despite his social anxiety, and had progressive improvements in his social skills. 

At the age of 13 years old, after his summer break, social isolation acutely worsened, associated with a confusional state, psychomotor agitation, speech impairment, visual hallucinations, cognitive regression, a loss of personal autonomy, and increased anxiety. Quetiapine up to 300 mg/day and alprazolam 0.50 mg/day were prescribed, with complete recovery. Cerebral MRI and metabolic tests were unremarkable. Array-CGH test was not significant, showing a duplication of the long arm of chromosome 6, inherited from the father. 

At the age of 15 years old, the patient had another acute breakdown, which was treated with quetiapine 300 mg/day and had partial recovery (only affective symptoms partly improved) until one year later, when symptoms worsened, with disorganized thought, obsessive symptoms and rumination, catatonic behaviors, associated with asthenia, reduced autonomous mobility, persistent hyporeactivity to stimuli, stiffness in the limbs and hypomymia, apathy, and isolation. Upon initial evaluation in the psychiatric ward, physical examination was unremarkable. Quetiapine was replaced with aripiprazole, with gradual titration, starting with 2.5 mg/day and 2.5 mg increases every 4 days, up to 10 mg/day, with supplementary lorazepam, resulting in a transient improvement in the clinical picture. After 2 days, the boy showed signs of psychomotor retardation, hyperreactivity to stimuli, anorexia, and asthenia. Creatine kinase (CK) was in the normal range when he was discharged. 

After 7 days, given the worsening symptoms associated with increased obsessive thoughts, hyperthermia, and CK elevation, the boy was admitted in an emergency department and pharmacotherapy was immediately discontinued. The patient was hospitalized in an intensive care unit for 4 weeks, then in a pediatric ward for 1 week, and finally in our hospital for 10 days. During hospitalization, limb stiffness, perioral myokymia and myoclonus, facial amimia, uncoordinated movements of the tongue and difficulty swallowing, polypnea, tachycardia, and arterial hypertension were observed. Intravenous hydration, dantrolene, clonidine, intravenous benzodiazepines, and carvedilol were administered, followed by bromocriptine therapy and intravenous lorazepam 2 mg 5 times a day. A gradual improvement in vigilance, reduction of hypertonus, and resolution of hyperthermia were observed, with gradual motor improvement. After 8 days, blood results showed a reduction in CK (404 U/L, normal CK range 0–50 UI/mL) and a mild increase in liver enzymes (ALT 72 U/L).

After being discharged from our hospital, the patient carried out monthly clinical and CPK controls, and after 6 months the patient had a general assessment in our hospital, with persisting control of previous clinical manifestations.

## 3. Discussion

In clinical practice, pharmacological treatments with antipsychotics are commonly used on individuals with ASD to improve associated symptoms such as irritability and agitation. The U.S. Food and Drug Administration and other European medical agencies have approved the use of aripiprazole for the treatment of irritability in autism, bipolar disorder, and schizophrenia in children and adolescents. Results from clinical studies on schizophrenia, bipolar disorder, conduct disorder, and comorbid autism show an increased risk of extrapyramidal symptoms when using atypical antipsychotics in children and adolescents, and an increase in akathisia as a common neurological adverse effect in these age groups [21]. The association between antipsychotic drug use on ASD patients and severe adverse events such as NMS is not well understood [22]. Our knowledge on NMS in the autistic pediatric population is still very limited, probably given the intrinsic difficulties of exploring NMS in controlled studies [23]. To the best of our knowledge, no review has ever been published focusing on NMS in an autistic pediatric population. When NMS is involved, case reports remain one of the main sources of information available in the literature. 

A challenging issue receiving increased attention, namely in ASD patients, is the relationship between NMS and catatonia. Catatonia is a mood and behavioral disorder characterized by a variety of symptoms such as stupor, catalepsy, waxy flexibility, mutism, negativism, posturing, and other symptoms [10]. Catatonia can occur in the context of a number of diseases, including psychotic and affective disorders, and neurodevelopmental disorders such as ASD seem to be particularly involved [24]. In a recent paper, Fink proposes to bring NMS, as well as other behavioral disorders (delirious mania, self-injurious behavior (SIB) in autism, and limbic encephalitis) within the catatonia tent [25]. This proposal is based on the observation of many common clinical features between those disorders and catatonia. In particular, there is a significant overlap of symptoms between NMS and malignant catatonia (MC), the most severe form of catatonia, associated with autonomic and thermoregulatory dysfunction [18,26]. Despite the two terms being sometimes used synonymously, the term NMS is used when there is a documented exposure to antipsychotic drugs. Additionally, NMS and other disorders under the catatonia tent respond well to catatonia treatments, which consist of a high-dose benzodiazepine as a first step, and electroconvulsive therapy (ECT) as a second step. Given these premises, recognizing catatonia assures lifesaving treatments [25]. In these case studies [18], every case of NMS was preceded by catatonic-like symptoms for weeks/months, fueling the ongoing debate about whether NMS is always preceded by catatonia, therefore representing a possible malignant form of catatonia triggered by antipsychotic assumption [27]. However, every patient apparently experienced worsened symptoms with antipsychotic administration after admission. 

In a review focused on the adverse events in children and adolescents treated with aripiprazole, the authors highlighted that, despite aripiprazole being considered safe in children, it may cause the same neurological adverse events, such as NMS, as in adults. Furthermore, ASD patients could also develop psychiatric adverse events [28]. Our patient presented a typical NMS clinical picture, confirming that the neuroleptic drugs could also cause NMS in children. 

A careful consideration of possible differential diagnoses should include all the conditions with prominent muscle rigidity and/or hyperthermia, such as malignant hyperthermia, malignant catatonia, and heath stroke. All the CNS infections should be ruled out (e.g., meningitis and encephalitis). Drug intoxication and toxicity (e.g., phencyclidine, ecstasy, cocaine, amphetamines, lithium, and baclofen) should also be considered. Serotonin syndrome, characterized by the presence of changes in mental status, agitation, clonus, hyperreflexia, and hyperthermia, with a degree of overlapping clinical presentation, may occur during serotonergic treatments, and the concurrent administration of an antidepressant with an antipsychotic drug may increase the risk of NMS due to serotoninergic transmission interfering with dopaminergic transmission. Progressive encephalomyelitis with rigidity and myoclonus (PERM), suggested to be a more severe variant of the stiff person syndrome, is a rare neurological condition, presenting an alteration of consciousness, cerebellar ataxia, and respiratory insufficiency due to breathing and swallowing difficulties [29]. 

Therefore, even though this risk should not prevent clinicians from using antipsychotics in severely impaired children and adolescents with ASD, early signs of NMS should be carefully monitored. A timely diagnosis of these signs may be more difficult to ascertain when ASD is severe, with mutism and stereotypies, particularly when intellectual disability co-occurs [29], thus in these patients the awareness of possible NMS is particularly important.

## Figures and Tables

**Table 1:** Case reports of pediatric ASD patients with neuroleptic malignant syndrome in the literature: demographic and clinical features.

	AGE	SEX	ASD	ID	AP	Preceding Catatonia	Other Symptoms	Reference
Patient 1	17	F	Yes	Yes	risperidone for 4.5 years, haloperidol and ziprasidone as needed	Yes	Restlessness, aggression, mutism, diaphoresis, sleep disturbances	Ghaziuddin et al., 2017 [18]
Patient 2	12	M	Yes	Yes	risperidone, quetiapine, ziprasidone, olanzapine	Yes	Restlessness, SIB, aggression, sleep disturbances	Ghaziuddin et al., 2017 [18]
Patient 3	15	F	Yes	Yes	ziprasidone, chlorpromazine	Yes	Posturing, aggression, mutism	Ghaziuddin et al., 2017 [18]
Patient 4	9	M	Yes	Yes	risperidone 1.5 mg/die, loxapine IM 30 mg/die	Yes	SIB, aggression, posturing, anorexia	González-Romero et al., 2019 [19]
Patient 5	16	M	Yes	Yes	aripiprazole 10 mg/die, quetiapine 250 mg/die, lorazepam	Yes	Anorexia, asthenia, perioral myokymia and myoclonus, difficulty swallowing, polypnea	This issue

Notes: ASD = autism spectrum disorder; ID = intellective disability; SIB = self-injurious behavior; AP = antipsychotic.

**Table 2:** Case reports of pediatric ASD patients with neuroleptic malignant syndrome in the literature: laboratory findings.

	Blood Pressure	Heart Rate	Fever	White Blood Count	AST (IU/L)	ALT (IU/L)	CPK (IU/L)	Cerebral MRI
Patient 1	Elevated	Elevated	Yes	Elevated	195 *	172 *	17,787 *	Normal
Patient 2	Elevated	Elevated	No	Normal	76 *	48 *	5993 *	Normal
Patient 3	Elevated	Elevated	No	NA	39	23	1800 *	MRI worrisome for vascular malformation, CT showed no evidence of AVM
Patient 4	NA	NA	Yes	NA	409 *	100 *	16,000 *	Normal
Patient 5	Elevated	Elevated	Yes	NA	NA	72 *	NA	Normal

Notes: * = abnormal laboratory finding; ALT = alanine transaminase; AST = aspartate transaminase; AVM = atrio ventricular malformation; CPK = creatinine phosphokinase enzyme; EEG = electroencephalogram; MRI = magnetic resonance imaging; NA = not available.

**Table 3:** Case reports of pediatric ASD patients with neuroleptic malignant syndrome in the literature: NMS duration, treatment, and outcomes.

	Nms Duration	Non Effective Tratments	Effective Treatments	Outcomes
Patient 1	Months-long	Lorazepam IV	ECT (total of 75 ECTs, during a 1 year mainteinance period)	Resolution, no relapses in 6 years after discontinuation of ECT
Patient 2	3 months	Lorazepam IV	ECT (3 years treatment duration)	2 relapses during previous attempts of discontinuation of ECT. Ultimately combined with lorazepam 32 mg/die, still ongoing
Patient 3	NA	Lorazepam IV	ECT (3 years treatment duration, still ongoing)	1 relapses during attempt of discontinuation of ECT
Patient 4	NA	Lorazepam IV	ECT (1 year treatment duration, still ongoing)	Positive outcome combined with behavioral therapy
Patient 5	17 days	/	Lorazepam IV, combined with dantrolene and bromocriptine	Resolution, no relapses in 2 years

Notes: NMS = neuroleptic malignant syndrome; NA = not available; ECT = electroconvulsive therapy.

## Data Availability

Not applicable.
